# Consumption of An Anthocyanin-Rich Antioxidant Juice Accelerates Recovery of Running Economy and Indirect Markers of Exercise-Induced Muscle Damage Following Downhill Running

**DOI:** 10.3390/nu11102274

**Published:** 2019-09-23

**Authors:** Leonardo C. R. Lima, Renan V. Barreto, Natália M. Bassan, Camila C. Greco, Benedito S. Denadai

**Affiliations:** 1 Human Performance Laboratory, São Paulo State University, Av. 24-A, 1515, Rio Claro, SP 13506-900, Brazil; 2 Centro Universitário Hermínio Ometto, Av. Dr. Maximiliano Baruto, 500, Araras, SP 13607-339, Brazil; 3 Centro Universitário Salesiano de São Paulo, R. Baronesa Geraldo de Resede, 330, Campinas, SP 13075-270, Brazil; 4 Centro Universitário UniMetrocamp, R. Dr. Sales de Oliveira, 1661, Campinas, SP 13035-500, Brazil

**Keywords:** running economy, antioxidant supplementation, anthocyanins, exercise-induced muscle damage, recovery, muscle soreness

## Abstract

This study examined the effects of anthocyanin-rich antioxidant juice (AJ) on the recovery of exercise-induced muscle damage (EIMD) and the running economy (RE) following downhill running (DHR). Thirty healthy young men were randomly divided into two blinded groups and consumed either AJ or placebo (PLA) for nine days (240 mL twice-a-day). On day 5, the participants from both groups ran downhill (−15%) for 30 min at 70% of their maximal oxygen uptake (VO_2_max) speeds. The changes in RE (oxygen uptake (VO_2_) and perceived effort (PE) during 5-min runs at 80%VO_2_max) and EIMD (isometric peak torque (IPT), muscle soreness (SOR) and serum creatine kinase activity (CK)) were compared over time and between the groups on the 4 days following DHR. VO_2_ and PE increased (*p* < 0.05) immediately following DHR for both groups and remained elevated for PLA until 48h post-DHR while fully recovering 24 h post-DHR for AJ. SOR was greater (*p* < 0.05) for PLA throughout the study. CK increased for both groups and was greater (*p* < 0.05) for PLA at 96 h post-DHR. IPT decreased for both groups but recovered faster for AJ (72 h) compared to PLA (no full recovery). AJ accelerated recovery of RE and EIMD and should be used in specific contexts, but not chronically.

## 1. Introduction

The running economy (RE) is an important predictor of performance in endurance events. It is defined as the amount of oxygen required to sustain running at a fixed submaximal speed [1]. RE represents, therefore, how efficient athletes are during running. Athletes with similar maximal oxygen consumptions (VO_2_max) may present different performances in long-distance runs due to the differences in RE [2].

Several factors influence RE acutely and chronically. A relationship between RE and neuromuscular aspects exists. A growing body of literature investigated the effects of exercise-induced muscle damage (EIMD) on parameters associated with RE [3,4,5]. EIMD occurs when muscle tissue is damaged following strenuous exercise, leading to compromised force production capacity, muscle soreness and leakage of intracellular proteins to the circulation [6].

Downhill running (DHR) has been reported as a damaging activity due to the high volume of eccentric contractions performed during the breaking phase of running associated with oxidative stress produced in the muscle by prolonged mitochondrial activity [3,4,7]. Assumpção et al. [8] have reviewed the literature and showed that DHR compromises RE as much as countermovement jumps and heavy-load squatting exercises [8]. 

Anthocyanins are phenolic compounds found in dark-colored fruits that act as a pigment in nature [9]. However, evidence suggests that anthocyanin rich foods have powerful antioxidant and anti-inflammatory properties when consumed by humans [10]. In fact, there is evidence that consuming anthocyanin-rich juices leads to faster recovery of markers of EIMD following resistance training [11] and endurance events [12]. The authors have recently reviewed the literature on the effects of consuming tart cherry juice (which is rich in anthocyanins and other phenolic compounds) in recovery from EIMD [13]. However, to the best of the authors’ knowledge, there are no studies investigating if the consumption of anthocyanin-rich juice accelerates the recovery of RE following damaging bouts.

The aim of the present study was to investigate the effects of anthocyanin-rich juice consumption on the magnitude of changes and time-course of recovery of markers of EIMD following a DHR bout. Our hypothesis was that the consumption of an antioxidant juice rich in anthocyanins would promote faster recovery of markers of EIMD and RE when compared to a placebo treatment. 

## 2. Materials and Methods

### 2.1. Participants

Thirty healthy male physical education students (age: 22.3 ± 2.6 years; height: 176.6 ± 6.4 cm; body mass 77.1 ± 10.5 kg) participated in the present study. The inclusion criteria for the present study were: Aged between 18 and 30 years-old; not having any experience with strength or aerobic training in the last six months; being a non-smoker and not having had lower-limb injuries in the last six months. They were instructed to refrain from intense physical activity, to keep their regular dietary habits and to drink plenty of water during the experimental period. All the participants read and signed an informed consent prior to their participation in the study, which was approved by the institution’s ethical board. A total of thirty participants were enrolled for the study and all of them completed the experimental protocol (15 per group). All the interactions and procedures in the present study were in accordance to the Declaration of Helsinki for research involving humans.

### 2.2. Experimental Design

The study was conducted under double-blind, placebo-controlled conditions. The participants were allocated to either experimental (EXP) or placebo (PLA) groups in a randomized fashion. Randomization was performed by the lead examiner using a draw application for smartphone. Before being assigned to each group, the participants were familiarized to the experimental procedures. The familiarization sessions included performing maximal isometric contractions on an isokinetic dynamometer (System 3, Biodex Systems, Shirley, NY, USA). The participants also had the opportunity to run for one minute on a treadmill (Pulsar, h/p/Cosmos, Germany) with 0% inclination to familiarize with treadmill running. In the second familiarization visit, the participants’ VO_2_max were determined.

After at least five days following the last familiarization session, the participants ran downhill (−15%) for 30 min at 70% of their VO_2_max speed. Chen et al. [3] showed that this protocol leads to significant damage to lower limb muscles and compromised RE. The knee extensors isometric peak torque (IPT), and markers of RE (oxygen uptake (VO_2_) and perceived effort (PE) during submaximal running bouts) were assessed 15 min before, 15 min after, and 1–4 days following DHR. Lower limb muscle soreness was assessed 15 min before and 1–4 days following DHR. Serum creatine kinase (CK) activity was assessed 15 min before, 2 and 4 days following DHR.

**Figure 1:**
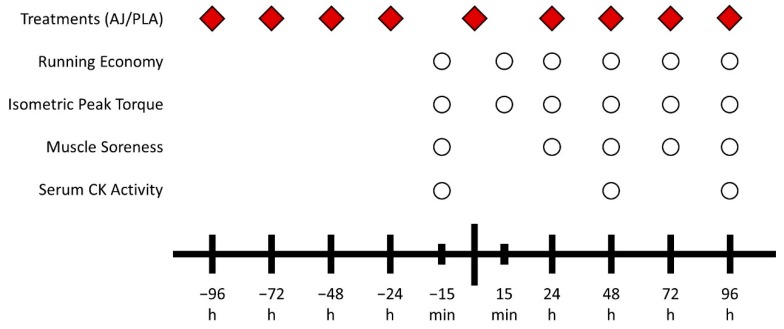
The experimental design of the study. The biggest dash represents downhill running. AJ: antioxidant juice; PLA: placebo; CK: creatine kinase.

The participants in the EXP group consumed 240 mL of an anthocyanin-rich antioxidant juice (Antiox, Juxx, Brazil) twice a day with a 12 h interval between doses at the day of the DHR bout and on the 4 days preceding and 4 days following it. Participants in the PLA group consumed a placebo consisting of water mixed with maltodextrin. The antioxidant juice and the placebo solution were isocaloric (106 Kcal per dose), isovolumetric (240 mL) and had the same amount of carbohydrates per dose (26 g). The experiment was conducted in a double-blinded fashion with all subjects and examiners blinded for the treatment being administered. The treatment bottles were opaque and the participants from both groups did not have contact with each other to avoid cross-contamination. The experimental design is illustrated in Figure 1.

### 2.3. Antioxidant Juice

The participants in the EXP group consumed an anthocyanin-rich antioxidant juice that consisted of a mixture of clarified apple juice with prum, blueberry, maquiberry, raspberry and cranberry concentrates. Each dose of the juice (240 mL) contained 58 mg of anthocyanins and an antioxidant capacity of 67,680 μmoL/mL of Trolox equivalent, as assessed by the oxygen radical absorbance capacity (ORAC5) scale to identify antioxidant capacity. The evidence suggests that consuming tart cherry juice with equivalent levels of anthocyanins attenuates muscle soreness and accelerates the recovery of muscle function following damaging bouts [11,12,14]. The daily intake and timing of antioxidant juice consumption in the present study were planned based on previous studies that showed enhanced recovery of indirect markers of EIMD due to the consumption of similar anthocyanin-rich juices [11,12,15,16].

### 2.4. Maximal Oxygen Uptake

VO_2_max was determined through a treadmill incremental test. The participants ran on a treadmill (Pulsar, h/p/Cosmos, Nussdorf-Traunstein, Germany) wearing a mask attached to a breath-by-breath gas analyzer (Quark PFT Ergo, Cosmed, Pavona, Italy) that recorded oxygen uptake (VO_2_) and carbon dioxide (CO_2_) production during exercise until test cessation. The incremental test started with a three-minute warm-up at 7 km/h followed by continuous 1 km/h increments every minute. The treadmill inclination was constant and set at 1%. The criteria adopted for cessation of the test were: (1) Heart rate of 95% of the predicted maximal (220-age); (2) the respiratory exchange ratio greater than 1,15; (3) voluntary fatigue. Following filtering of the data, VO_2_max was considered as the greatest VO_2_ value recorded and sustained for at least 15 s during the test. The speed at which VO_2_max was reached (sVO_2_max) was also recorded.

### 2.5. Running Economy

The running economy was assessed at a fixed-speed of 5 min runs at 80% of individual sVO_2_max. This intensity was chosen based on the findings of Chen et al. [4] that RE at 80% and 90% sVO_2_max, but not at 70% sVO_2_max, is compromised following DHR. Therefore, this study adopted the lowest intensity at each RE which is compromised following DHR. The breath-to-breath gas exchanges were registered during RE tests and the mean VO_2_ was recorded during the fifth minute of each test. At the end of each RE test, the participants rated their perceived effort in a scale that varied from 6 to 20 [17]. The VO_2_ at the last minute of the RE tests and the perception of effort were recorded as metabolic and perceptual indices of RE, respectively. 

### 2.6. Indirect Markers of Exercise-Induced Muscle Damage

IPT, muscle soreness and serum CK activity were assessed as indirect markers of EIMD. To assess IPT, the participants performed two 5 s maximal voluntary isometric contractions in an isokinetic dynamometer with a 180 s recovery interval between contractions. A signal acquisition device with a sampling frequency of 1000 Hz (Miotool, 200/400, Miotec, Porto Alegre, Brazil) was synchronized with the dynamometer (System 3, Biodex Systems, Shirley, NY, USA) during the maximal voluntary isometric contractions for greater precision during data acquisition. The participants were seated at the dynamometer following the manufacturer’s guidelines, with their trunks, hips and dominant thighs firmly secured to the chair, their knees flexed at 70°, and their legs firmly attached to the dynamometer shaft. They were instructed to perform knee extensions as quickly and forcefully as possible during 5 s with strong verbal encouragement being provided by the examiners. The acquired data was saved and stored for subsequent analyses.

The data obtained during the maximal voluntary isometric contractions was filtered (Butterworth filter, low pass, 4th order, with a 15 Hz cut-off frequency) and analyzed in a MatLab environment (MatLab 6.5, Mathworks, Natick, MA, USA). IPT was considered as the greatest value in the torque-time curve. The contraction with the greatest IPT was used for further analyses.

Muscle soreness was quantified using a 1000 mm visual analogs scale with the saying “not sore at all” and “very, very sore” at the extremities. The participants were instructed to rate their perceived soreness after climbing up and down from a 45 cm chair with their dominant limb without external assistance. They performed this test following 5 min of seated rest and could perform as many repetitions as necessary. They were instructed to mark the visual analogs scale according to the soreness they felt on their knee extensors after completing the stepping exercise.

Serum CK activity was quantified by spectrophotometric analyses. Further, 500 μL of blood was extracted from the participant’s earlobes 5 min after the application of a vasodilator ointment (Finalgon, Pharma GmbH & Co. KG, Aachen, Germany) to avoid hemolysis. The blood samples were allowed to clot for 10 min and centrifuged for 10 min at 56,000 rpm (Microhemato, Modelo 2410, Fanem, São Paulo, Brazil) and the serum samples were extracted and stored at −70 °C for further analyses. Serum CK activity was determined using a commercial kit (CK-NAC UV, Wiener Lab, Rosário, Argentina) and a spectrophotometer (Bio-2000, Bioplus, São Paulo, Brazil). The reference values for healthy men for the method used ranged between 24 and 195 U/l.

### 2.7. Statistics

Data normality was confirmed using the Shapiro-Wilk test. Data sphericity and homogeneity were confirmed using the Mauchly and Levene tests, respectively. The differences between groups in baseline values for all dependent variables as well as anthropometric data were tested by the student’s *t*-test. The changes over time and between groups were compared using the mixed model ANOVAs for repeated (time) and non-repeated (groups) measures with Bonferroni post-hoc tests. All analyses were performed in a professional software (Statistical Package for Social Sciences 17, IBM, Armonk, NY, USA). The significance levels were set at *p* <0.05. The data are expressed as the means ± standard deviation unless otherwise stated.

## 3. Results

The participants’ mean age, body mass, height, body mass index, VO_2_max, sVO_2_max, DHR speeds and RE-test speeds are presented in Table 1. No significant differences in such variables were found. 

No significant differences between groups were found for baseline values of VO_2_ (CON: 33 ± 3.8 mL·kg^−1^·min^−1^; EXP: 35.1 ± 3.3 mL·kg^−1^·min^−1^), perceived effort (CON: 11.5 ± 1.2; EXP: 12.3 ± 1.2), IPT (CON: 290 ± 34 Nm; EXP: 278 ± 36 Nm), knee extensor muscle soreness (CON: 0 ± 0 mm; EXP: 0 ± 0 mm) and serum CK activity (CON: 106 ± 49 U.l^−1^; EXP: 126 ± 40 U.l^−1^). The significant group versus the time interactions were found for VO_2_ (F(5) = 20.05, *p* < 0.01), perceived effort (F(5) = 4.86, *p* < 0.01), IPT (F(5) = 3.80, *p* = 0.003), knee extensor muscle soreness (F(4) = 3.82, *p* < 0.01) and serum CK activity (F(2) = 3.70, *p* = 0.31).

**Figure 2:**
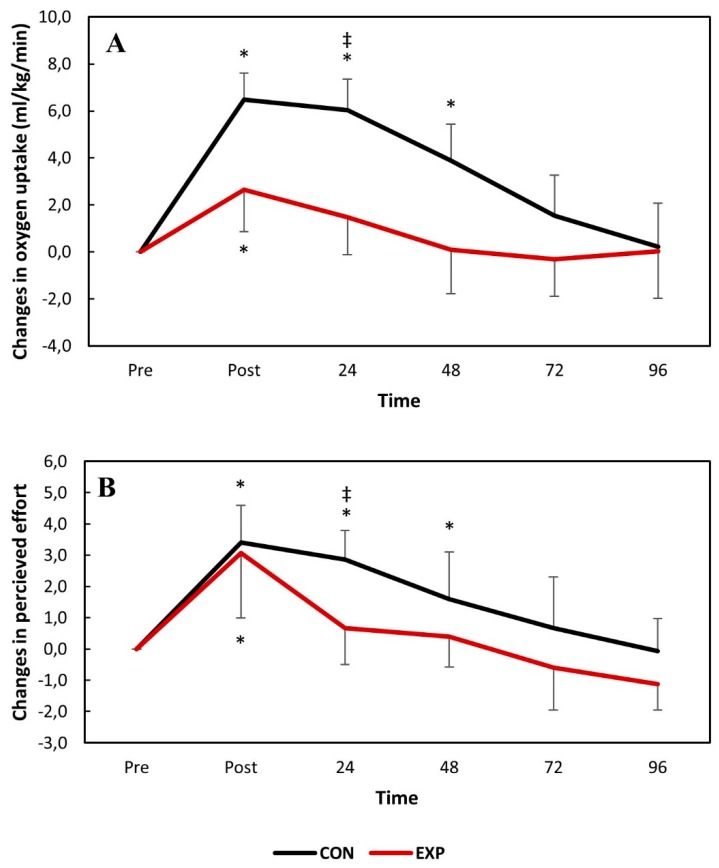
The absolute changes in oxygen uptake (**A**) and perceived effort (**B**) over time following downhill running. * *p* < 0.05 compared to the baseline values in the same group. ^‡^ *p* < 0.05 compared to the experimental group at the same time-point. CON: control group; EXP: experimental group.

The pairwise comparisons showed that VO_2_ significantly increased for both groups immediately following DHR and fully recovered 24 h and 72 h post-exercise for the experimental and control groups, respectively. VO_2_ was significantly greater for the control group than the experimental group 24 h post-DHR. This was also the case for perceived effort. The absolute changes in VO_2_ and perceived effort over time following DHR are presented in Figure 2.

**Figure 3:**
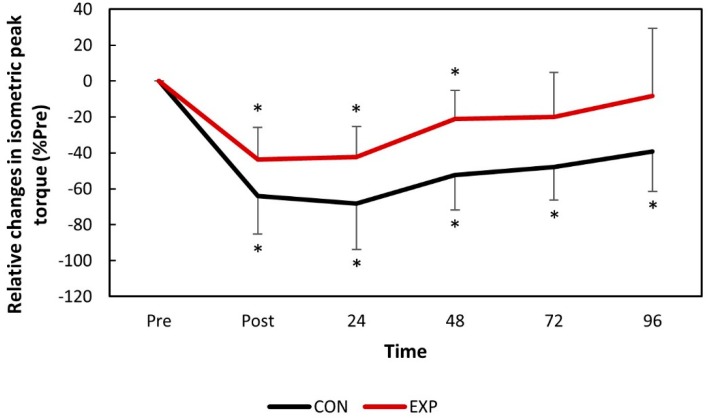
The relative changes in isometric peak torque over time following downhill running. * *p* <0.05 compared to the baseline values in the same group. CON: control group; EXP: experimental group.

The isometric peak torque significantly decreased (*p* < 0.05) for both groups immediately after DHR and remained so throughout the entire experimental period for the control group, but fully recovered 72 h post-DHR for the experimental group (Figure 3).

**Figure 4:**
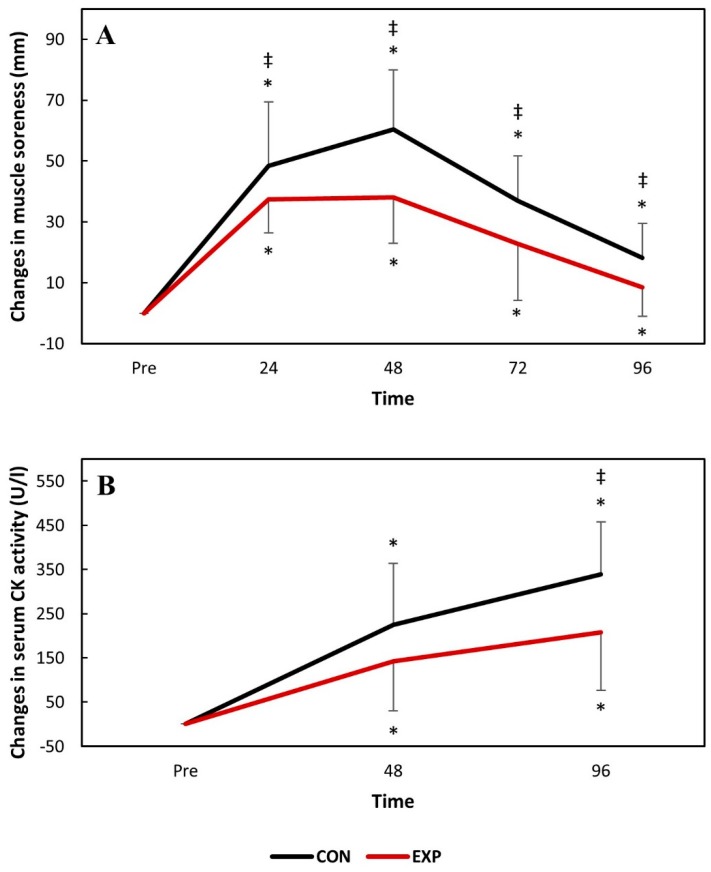
The changes in knee extensor muscle soreness (A) and serum creatine kinase (CK) activity (B) over time following downhill running. * *p* <0.05 compared to baseline values in the same group; ^‡^ *p* <0.05 compared to the experimental group at the same time-point. CON: control group; EXP: experimental group.

Knee extensor muscle soreness significantly increased (*p* < 0.05) 24 h following DHR and remained so during the whole experiment for both groups. However, knee extensor muscle soreness was significantly (*p* < 0.05) greater for the control group at all time-points. Serum CK activity significantly increased 48 h following DHR and remained elevated until 96 h following DHR for both groups. Serum CK activity was significantly (*p* < 0.05) greater for the control group at 96 h post-DHR. The changes in knee extensor muscle soreness and serum CK activity are presented in Figure 4.

## 4. Discussion

The aim of the present study was to investigate the acute impact of antioxidant juice consumption on changes in RE and the recovery of indirect markers of EIMD following DHR. It was hypothesized that phenolic compounds present in the antioxidant juice—especially anthocyanins—would accelerate recovery of muscle soreness, serum CK activity, muscle function and RE following DHR, and this was confirmed by the obtained data.

The data from the control group showed that DHR significantly compromises RE at 80% sVO_2_max—measured as VO_2_ and the perceived effort—with full recovery reached 3 days following the exercise bout. DHR also led to significant changes in the indirect markers of EIMD (IPT, muscle soreness and serum CK activity) with full recovery not being reached within four days following the exercise bout for the control group. These findings corroborate what has been previously reported in the literature and confirm that recovery kinetics are different between RE and the indirect markers of EIMD [8].

The present study found that antioxidant juice consumption results in faster recovery of muscle function as well as attenuated muscle soreness and serum CK activity following an exercise bout consisting of 30 min of DHR. Our findings are similar to those which showed that consuming tart cherry (*Prunus cerasus* L.) juice accelerates recovery of the indirect markers of EIMD following the different types of exercise bouts (i.e., resistance exercise training, maximal isokinetic eccentric contractions, DHR, marathon running and stochastic cycling) [11,12,16,18,19].

However, little is known about the mechanisms underlying downhill running-induced muscle damage. It is generally accepted that EIMD is characterized by two distinct events. In the first event, the mechanical strain imposed by unaccustomed exercise damages the sarcolemma and ultrastructural sarcomere proteins [20]. This results in compromised muscle function due to the disrupted contractile and structural proteins as well as the compromised excitation-contraction coupling [6]. The mechanical damaging event is usually aggravated when eccentric contractions are performed during unaccustomed exercise bouts due to the unique motor unit recruitment patterns during such contractions [21]. 

The mechanical damage is followed by cellular signaling for repair, which triggers an inflammatory response consisting of the migration of neutrophils and monocytes (which differentiate into macrophages once in the damaged site) [22]. The immune cells promote the degradation of cellular debris through phagocytosis by producing oxygen reactive species. However, this degradation is not exclusive to cellular debris, but also affects healthy, functioning, structures of adjacent myocytes. This is referred to as the second event of EIMD and leads to muscle soreness, increased CK release to the blood stream and, possibly, further loss of muscle function [13]. It is yet to be determined if the oxidative stress produced by the mitochondrial respiratory chain during DHR anticipates and/or aggravates the second event of EIMD. It is, however, well established that DHR significantly affects the indirect markers of EIMD [8,23]. 

The data obtained in the present study suggests that muscle function was significantly compromised immediately following DHR for both groups. This was expected, since the consumption of antioxidant juice is not expected to strengthen the sarcolemma nor impact the motor unit recruitment patterns, attenuating the first EIMD event. However, accelerated recovery kinetics were observed for both RE (VO_2_ and perceived effort) and muscle function (IPT) (Figure 2 and Figure 3). The perceived effort and VO_2_ fully recovered two days earlier for the experimental group with significant differences between the groups observed 1 day following DHR. Similarly, IPT reached full recovery during the study in the experimental group while it remained compromised throughout the entire study for the control group. No significant differences were found between the groups for the IPT values. Although previous studies have reported attenuated changes in IPT following damaging bouts when associated with consumption of tart cherry juice [11], accelerated recovery kinetics are also important when investigating strategies to attenuate EIMD [16,24].

The differences between changes in RE and muscle function observed among the groups in the present study might be explained by the antioxidant properties of anthocyanins in the antioxidant juice. Previous studies showed that anthocynin-rich tart cherry juice reduced total oxidative stress and circulating levels of C-reactive protein other oxygen reactive species [12,16,25]. It has been reported that anthocyanins (as well as other phenolic compounds) scavenge free radicals secreted by lymphocytes and produced in the mitochondrial respiratory chain [26].

It has also been reported that consuming foods as rich in anthocyanins as the antioxidant juice used in the present study decreases circulating levels of pro-inflammatory cytokines such as interleukin-6 and tumor necrosis factor-α following damaging bouts [12,19], potentially attenuating the second event of EIMD. This might also explain the attenuated muscle soreness for the experimental group observed at all assessment points in our study. The delayed-onset muscle soreness is frequently described as a symptom of the inflammation that occurs in the muscle and fascia following eccentric-biased activities due to the interaction of algesic pro-inflammatory substances such as histamines, bradykinins and prostaglandins with nociceptors [27]. Hence, if a treatment attenuates inflammation, it also attenuates the ensuing soreness caused by it.

Serum CK activity increased for both groups following downhill running, peaking 4 days after it. However, serum CK activity was greater for the control group at its peak. As an intracellular enzyme, the increased CK activity in the bloodstream is a sign of membrane and tissue damage [28]. Peak CK activity in the bloodstream occurs later than other indirect markers of EIMD since it must be transported from the lymph to the circulation [28]. Significantly greater serum CK activity for the control group suggests either greater mechanical stress (which was not the case, since both groups exercised at identical volumes and intensities) or that membrane damage caused by lipolytic enzymes such as phospholipase A2—which is activated by pro-inflammatory cytokines [29]—was greater in the absence of the treatment investigated in the present study. It can, therefore, be assumed that the anti-inflammatory properties of the treatment investigated in the present study attenuated secondary damage to healthy myocytes induced by inflammation.

Although oxidative-stress or inflammatory markers were not assessed in the present study, it is important to notice that the concentration of anthocyanins in the antioxidant juiced consumed by the participants in the experimental group is similar to those of cherry juices used in studies that found attenuated inflammatory responses and total oxidative status. Associated with the observation of similar effects on the indirect markers of EIMD between our treatment and those previously reported, this adds to the presented rationale.

To the best of the authors’ knowledge, no previous study investigated the impact of consuming antioxidant/anti-inflammatory treatments in the magnitude of changes and recovery kinetics of RE following damaging bouts. This study found that not only does consuming antioxidant juice accelerate recovery of RE markers, but it also attenuates changes 1 day following DHR. In fact, our results suggest that RE is only compromised immediately following DHR when consuming antioxidant juice. This has important implications regarding training protocols and, especially, competitive schedules. 

In specific contexts, athletes are submitted to short-term competitions at which they are expected to perform in subsequent days. In such contexts, it is important for endurance athletes to maintain their efficiency, and EIMD from previous days might be an issue. Training camps are also an example of condensed endurance events during which it is of the best interest of athletes to perform as well as possible. Our findings indicate that consuming an antioxidant juice rich in anthocyanins might be a good strategy to maintain efficiency and attenuate muscle soreness in such contexts. Caution is warranted when transferring the finding of the present study to trained athletes. The participants in our study presented VO_2_max values between 40−45 mL·kg^−1^·min^−1^, which are not compatible with trained athletes. The evidence suggests that antioxidant status is greater for athletes compared to sedentary controls [30]. Hence, there is a possibility that additional antioxidant properties of anthocyanin-rich foods do not further improve the already-high antioxidant response to damaging exercises in trained athletes. However, Howatson et al. [12] showed that the consumption of *Prunus cersasus* L. accelerates recovery of muscle function and soreness following marathon running in experienced runners. Further studies are warranted to investigate if anthocyanin-rich foods accelerate recovery of RE in elite athletes.

The continuous use of antioxidant juices during endurance training programs should not be encouraged. The evidence suggests that, despite the beneficial effects of antioxidant supplementation in the recovery from EIMD and RE, oxidative stress might be an important component for training adaptation [31]. Merry and Ristow [32] reviewed the literature on this topic and concluded that a balance in redox signaling is the key to optimal endurance adaptation and long-term antioxidant supplementation can blunt the physiological stress imposed by exercise, consequently compromising optimal training adaptation. This should be taken into consideration when planning nutritional strategies for training and competitions. 

The assessment of oxidative status and markers of inflammation is important, and the absence of these variables is a limitation of our study. While this study did find a significant impact of the treatment in our main outcome, the direct markers of secondary damage to better elucidate the mechanisms of faster RE recovery associated with antioxidant juice consumption were not assessed. The authors encourage further investigation of such mechanisms. This study also did not carry out dietary analyses. This might have implications regarding an already high antioxidant status due to the high anthocyanin intake by the participants prior to the experiment. Since this study only recommended the participants to maintain their regular dietary habits but did not control nor assess them during the experimental period, this might also be considered a limitation of the present study. Another limitation of the present study is the intensity at which RE was assessed. Future studies should assess the impacts of antioxidant juice consumption on the changes in RE at different intensities following DHR and other damaging bouts. Finally, there seems to be a lack of standardization for the composition of antioxidant products investigated in the literature. this study focused on anthocyanin concentration and antioxidant capacity when choosing our treatment, as well as the commercial availability in our country. Therefore, the findings presented in the present study represent the impact of this specific antioxidant juice on changes in RE and the indirect markers of EIMD, which may not be the same when using antioxidant juices with other compositions. It is recommended that practitioners and colleagues focus on anthocyanin concentration when choosing which juice to prescribe/investigate.

## 5. Conclusions

In conclusion, this study shows that consuming an anthocyanin-rich antioxidant juice four days prior to, at the day and four days following DHR resulted in the accelerated recovery of RE and muscle function as well as attenuated muscle soreness. These data suggest that this nutritional strategy might be useful to maintain satisfactory performance in condensed competitions and training camps. Caution is warranted when planning long-term antioxidant supplementation, as training adaptations might be blunted. Future studies are needed to clarify the mechanisms underlying faster recovery of RE when consuming antioxidant juice.

## Figures and Tables

**Table 1:** The characteristics of the sample for the experimental (EXP) and placebo (PLA) groups.

	PLA (*n* = 15)	EXP (*n* = 15)
Age (years)	22.8 ± 2.8	21.9 ± 2.3
Body mass (kg)	79.5 ± 11.8	74.6 ± 8.7
Height (m)	1.74 ± 0.07	1.77 ± 0.06
BMI (kg/m^2^)	26.2 ± 3.2	23.7 ± 2.2
VO_2_max (mL/kg/min)	41.8 ± 5.7	43.7 ± 4.3
sVO_2_max (km/h)	13.9 ± 1.4	14.7 ± 1.2
Downhill Running Speed (km/h)	9.7 ± 1	10.3 ± 0.9
Running Economy Test Speed (km/h)	10.1 ± 1.2	10.5 ± 1

PLA: Placebo group; EXP: Experimental group; BMI: Body mass index; VO_2_max: Maximal oxygen uptake; sVO_2_max: Speed at which the maximal oxygen uptake was achieved.
